# Comparison of In Vitro Metrics With Real-World Risk of Drug-Induced Parkinsonism Due to Antipsychotic Drugs: Retrospective Cohort Study

**DOI:** 10.2196/81876

**Published:** 2026-01-28

**Authors:** Woo-Taek Lim, Hyun Woo Lee, Seungyeon Kim, Kwangsoo Kim, Yong Min Ahn, Minseok Hong, Yun Mi Yu, Ha Young Jang

**Affiliations:** 1 College of Pharmacy, Gachon University, 191, Hambangmoe-ro, Yeonsu-gu, Incheon, 21936, Republic of Korea, +82-32-820-4937; 2 Department of Pharmaceutical Medicine and Regulatory Sciences, Colleges of Medicine and Pharmacy, Yonsei University, Incheon, Republic of Korea; 3 Department of Pharmacy and Yonsei Institute of Pharmaceutical Sciences, College of Pharmacy, Yonsei University, Incheon, Seoul, Republic of Korea; 4 College of Pharmacy, Dankook University, Cheonan, Republic of Korea; 5 Division of Clinical Bioinformatics, Biomedical Research Institute, Seoul National University Hospital, Seoul, Republic of Korea; 6 Department of Psychiatry, Seoul National University Hospital, Seoul, Republic of Korea; 7 Department of Psychiatry, Uijeongbu Eulji Medical Center, Eulji University, Uijeongbu, Republic of Korea

**Keywords:** drug-induced parkinsonism, antipsychotic drug, binding affinity, dopamine D2 receptor, blood-brain barrier

## Abstract

**Background:**

Drug-induced parkinsonism (DIP) predominantly occurs due to antipsychotic drugs (APDs) blocking dopamine D2 receptors (D_2_Rs). However, in vitro assays often fail to fully reflect real-world variability in clinical outcomes.

**Objective:**

This study aimed to evaluate whether in vitro pharmacological metrics correspond to real-world risk of DIP associated with APD use.

**Methods:**

For 8 commonly used APDs, key in vitro parameters—including inhibition constants (K_i_) of D_2_Rs and the serotonin 2A receptor, reversal rate (K_r_) of D_2_Rs, and blood-brain barrier (BBB) penetration rate—were compiled to construct 6 composite DIP risk metrics. The real-world DIP risk was assessed using the Seoul National University Hospital common data model (2002‐2021). APD users were matched 1:1 to selective serotonin reuptake inhibitor users using propensity score matching, and Cox proportional hazard regression was performed to estimate the hazard ratios (HRs) for DIP risk. Correlation between each in vitro metric and real-world DIP risk was evaluated using logarithmic regression models.

**Results:**

Among 44,664 patients from 8 matched cohorts, haloperidol showed the highest DIP risk (HR=4.56, 95% CI 2.29‐9.07), whereas aripiprazole exhibited the lowest risk (HR=2.11, 95% CI 1.56‐2.86). Metric 4 (pK_r_ × BBB penetration rate) exhibited the strongest correlation with real-world DIP risk (*R*^2^=0.95). The correlation decreased when aripiprazole, a partial D_2_R agonist, was included in the analysis (*R*^2^=0.58).

**Conclusions:**

Integrating receptor-binding kinetics with BBB penetration may provide an in vitro framework that reflects real-world variation in DIP risk among D_2_R-antagonizing APDs. These findings support the relevance of combining kinetic and central nervous system exposure parameters for early safety evaluation.

## Introduction

The pharmacological action of drugs generally occurs through receptor binding, and side effects are often the consequences of these interactions [1]. To understand how drugs function and anticipate their potential side effects, extensive in vitro experiments on drug-receptor interactions are conducted from the early stages of drug development. However, relying on in vitro analysis, often referred to as “test tube experiments,” makes it difficult to predict how often side effects will occur in real-world patients [2,3]. This translational gap has highlighted the need for mechanistically informed models that integrate binding kinetics and pharmacokinetic factors to better approximate real-world safety outcomes [3].

Drug-induced parkinsonism (DIP), predominantly caused by antipsychotic drugs (APDs), is one of the most common forms of secondary parkinsonism and remains a clinically significant dose-limiting adverse effect [4]. DIP arises primarily from dopamine D2 receptor (D_2_R) blockade within the nigrostriatal pathway, leading to symptoms of movement disorders such as muscle stiffness, slow movements, and tremors [5-10]. Because these symptoms can impair quality of life and daily functioning, understanding the pharmacologic determinants that underlie variability in DIP risk across APDs has become an important clinical and research priority [11,12].

In efforts to explain interdrug differences, the inhibition constant (K_i_) and dissociation constant (K_d_) have been widely used as a key measure for D_2_R blockade; lower values indicate stronger binding to D_2_Rs at equivalent drug concentrations [13]. Sykes et al [14] further expanded this framework by demonstrating that receptor-rebinding kinetics—reflecting the probability that a ligand re-engages adjacent receptors after dissociation—can also account for differences in extrapyramidal symptom liability. Other studies have implicated the serotonin 2A receptor (5-HT_2A_R) in DIP modulation, with newer APDs often exhibiting a higher 5-HT_2A_R–to-D_2_R affinity ratio associated with lower DIP risk [15-17]. While binding kinetics contributes to understanding variation in APD-related neurological syndromes, additional pharmacological factors also appear to play important roles. A recent meta-analysis demonstrated that the dose of APDs and D_2_R occupancy correlate with extrapyramidal symptom onset, indicating that in vivo exposure should be considered alongside in vitro parameters [18,19]. Furthermore, blood-brain barrier (BBB) permeability, which regulates central nervous system (CNS) drug exposure and can change with certain clinical conditions, may also influence the clinical expression of D_2_R blockade [20].

**Figure 1.**
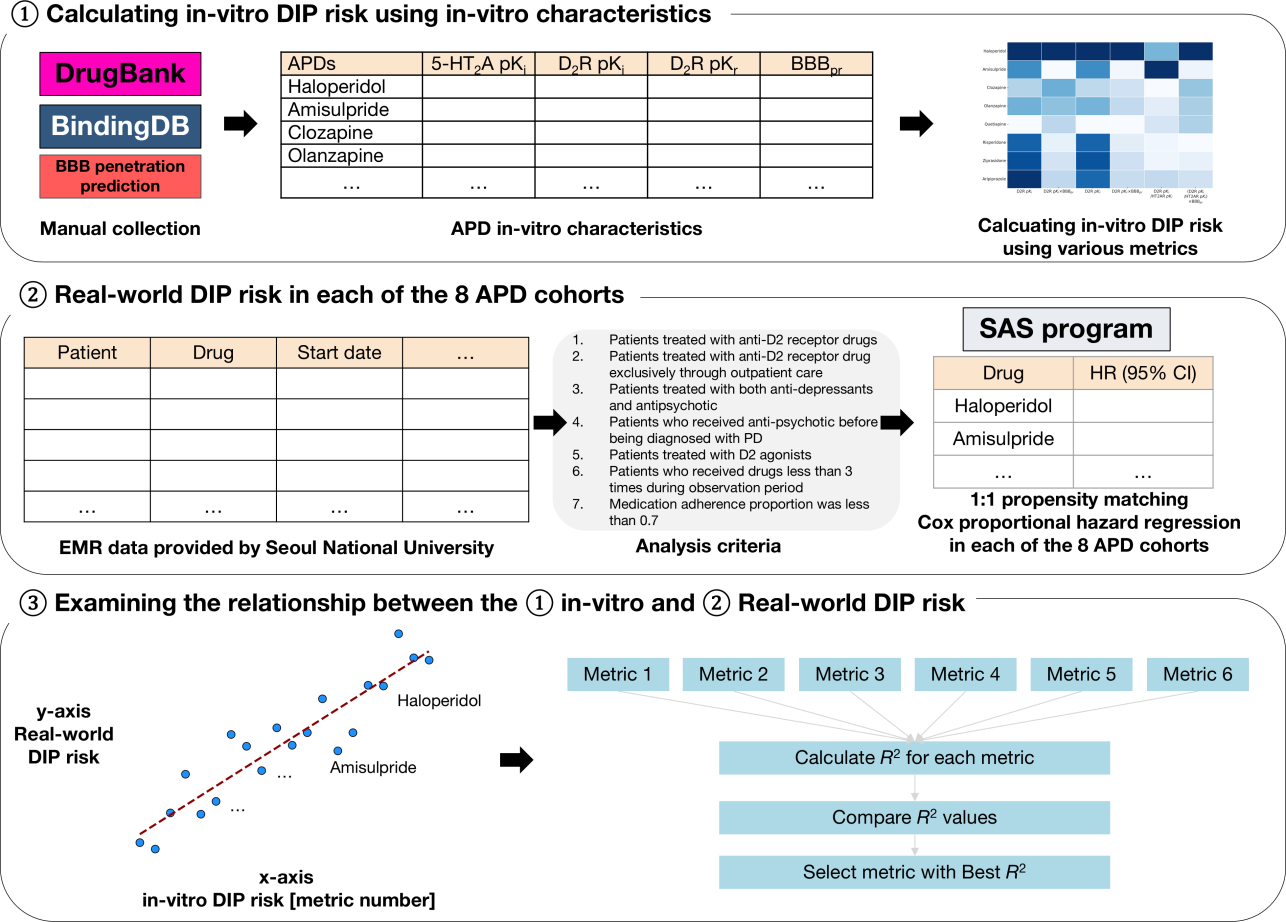
Study overview. 5-HT_2A_R: serotonin 2A receptor; APD: antipsychotic drug; BBB_pr_: blood-brain barrier penetration rate; D_2_R: dopamine D2 receptor; DIP: drug-induced parkinsonism; EMR: electronic medical record; HR: hazard ratio; PD: Parkinson disease.

These prior observations collectively suggest that DIP risk reflects an interplay among receptor-binding kinetics, CNS pharmacokinetics, and dose-response. However, despite extensive research on DIP, its precise mechanism and the quantitative translation between in vitro pharmacological parameters and real-world DIP risk remain unclear [6,7,21]. Therefore, this study aimed to evaluate whether specific in vitro pharmacological metrics correspond to variation in real-world DIP risk, with a particular focus on D_2_R-antagonizing APDs. First, we derived multiple candidate in vitro DIP risk metrics based on receptor-binding and pharmacokinetic parameters of APDs (Figure 1). Second, using longitudinal real-world data (RWD), we estimated the DIP risk associated with commonly used APDs. Finally, we assessed the extent to which each in vitro metric correlated with the observed real-world DIP risk.

## Methods

### Study Design and Overview

This study consisted of two components: (1) an assessment of in vitro pharmacological parameters of APDs related to DIP and (2) a retrospective cohort study evaluating the effect of APDs on the risk of DIP using the common data model (CDM) of Seoul National University Hospital. We subsequently examined the relationship between in vitro metrics and the real-world DIP risk. This study was reported in accordance with the Strengthening the Reporting of Observational Studies in Epidemiology guidelines [22].

### In Vitro DIP Risk Metrics

The key parameters of the APDs used to calculate the in vitro DIP risk were the pK_r_ values for D_2_Rs and 5-HT_2A_R collected from DrugBank and BindingDB [23-25]. The receptor reversal rate (K_r_) values for D_2_Rs as suggested by Sykes et al [14] were also obtained. Considering that the primary site of action for APDs is within the CNS, the BBB penetration rate of each APD was considered an adjustment factor [26]. The 6 main metrics for the in vitro DIP risk were as follows:

- Metric 1: pK_i_ for D_2_Rs
- Metric 2: (pK_i_ for D_2_Rs) × BBB penetration rate
- Metric 3: pK_r_ for D_2_Rs
- Metric 4: (pK_r_ for D_2_Rs) × BBB penetration rate
- Metric 5: (pK_i_ for D_2_Rs) / (pK_i_ for 5-HT_2A_R)
- Metric 6: (pK_i_ for D_2_Rs) / (pK_i_ for 5-HT_2A_R) × BBB penetration rate

All the data were collected through a manual search conducted by the research team. When multiple values were reported, the geometric mean was used as an integrated value.

### RWD Source

The CDM of Seoul National University Hospital included longitudinal data on patient demographics, diagnostic information (such as Parkinson disease and other comorbidities), and prescription details (including prescribed drugs, prescription dates, dosages, and duration of use) from 2002 to 2021. The CDM includes standardized fields for these domains, so conventional item-level missingness is minimal; however, care and prescriptions received outside the hospital are not captured and may result in incomplete ascertainment of medication exposure and clinical events, which was considered when interpreting the results.

### Study Population

Patients were recruited into the study cohort based on prescription records. The experimental group consisted of patients prescribed APDs, including haloperidol, olanzapine, quetiapine, risperidone, amisulpride, aripiprazole, clozapine, and ziprasidone. The active comparator group, which served as the control, consisted of patients treated with selective serotonin reuptake inhibitors (SSRIs), including citalopram and escitalopram, fluoxetine, paroxetine, and sertraline, which are known to have minimal D_2_R binding affinity (K_i_ of approximately 10,000 nM) [23-25].

The index date was defined as the date of the first outpatient prescription of an APD or an SSRI during the study period. To maintain methodological consistency, only outpatient prescriptions of the oral formulations of the study drugs were included in the analysis. Patients were excluded based on the following criteria: (1) concurrent prescriptions of APDs and SSRIs; (2) diagnosis of Parkinson disease prior to the index date as the study focused on identifying new-onset DIP attributable to drug exposure; (3) use of D_2_R agonists within 1 year prior to the index date; (4) fewer than 3 prescriptions of the study drugs after the index date because such limited exposure is unlikely to represent sustained treatment and may not meaningfully influence DIP onset (in our setting, outpatient prescriptions of APDs and SSRIs are usually written for short durations [approximately 2‐4 weeks], so at least 3 prescriptions typically correspond to 2 to 3 months of continuous therapy and help avoid misclassifying sporadic or trial use as ongoing treatment, consistent with previous register-based studies evaluating psychotropic and antipsychotic medication exposure [27,28]); and (5) poor medication adherence, defined as a proportion of days covered of less than 0.7 as inconsistent medication use could confound assessments of DIP risk [29].

### Outcome Assessment

The onset of DIP was defined by the presence of diagnostic codes for DIP (G21.1, G21.2, G21.8, and G21.9) based on the *International Classification of Diseases, 10th Revision* [30]. To enhance diagnostic specificity, patients were considered to have developed DIP only if they received prescriptions for D_2_R agonists or anticholinergic agents (standard treatments for DIP) within 60 days of initial diagnosis.

### Covariates

A total of 28 covariates were selected based on the presence of comorbidities or concurrent medication history within 1 year of the start date of medication use. The 11 comorbidities included chronic obstructive pulmonary disease, dementia, diabetes mellitus, dyslipidemia, end-stage renal disease, gout, hypertension, liver disease, osteoarthritis, osteoporosis, and stroke. The list of *International Classification of Diseases, 10th Revision* codes for comorbidities is shown in Table S1 in Multimedia Appendix 1. The 17 concurrent medications included renin-angiotensin-aldosterone system inhibitors, such as angiotensin-converting enzyme inhibitors and angiotensin receptor blockers; alpha-glucosidase inhibitors; anticonvulsants; anxiolytics; beta-blockers; calcium channel blockers; dipeptidyl peptidase-4 inhibitors; erythropoiesis-stimulating agents; glucagonlike peptide-1 receptor agonists; insulin; iron; loop diuretics; meglitinides; metformin; sodium-glucose cotransporter 2 inhibitors; statins; and sulfonylureas. A detailed list of concurrent medications is provided in Table S2 in Multimedia Appendix 1.

### Statistical Analysis

Statistical analyses were performed for the treated population. Patients were followed up on until the earliest events of DIP onset, the last day the patient took their prescribed medication + 30 days, or the end of the 1-year study period. Each APD user was matched 1:1 to an SSRI user, and the distribution of the propensity score was inspected [31]. The matching variables included age, sex, comorbidities, and concurrent medications. A standardized difference of >0.1 was regarded as a sign of imbalance [32]. The baseline characteristics were summarized using descriptive statistics. Cox proportional hazard regression was used to estimate the hazard ratio (HR) and 95% CI for the risk of DIP associated with APD use. A dose-response analysis was performed by stratifying patients according to their average daily exposure based on the defined daily dose (DDD) [33]. Patients were categorized into 3 groups (<0.5 the DDD, 0.5‐1.5 the DDD, and ≥1.5 the DDD) to contextualize the in vitro correlation within a clinical dose-response pattern.

### Correlation Analysis

To explore the relationship between the in vitro and real-world DIP risks, we correlated the 6 in vitro metrics with the HR estimated from the real-world cohort. Coefficients of determination (*R*^2^) were calculated to quantify the explanatory strength of each association, and an *R*^2^ value of 0.7 was used as a descriptive threshold for a strong relationship [34]. The primary analysis focused on APDs with D_2_R antagonist properties, whereas aripiprazole—a partial agonist—was examined separately in an exploratory analysis to reflect its distinct pharmacologic profile [35].

For each drug i, the association between the clinical outcome—expressed as an HR—and the corresponding in vitro pharmacological metric (metric 1‐6) was modeled using a logarithmic regression:


$$HR_{i}=\beta_{0}+\beta_{1}\ln\left(X_{i}\right)+\varepsilon_{i}$$

In this expression, $X_{i}$ represents the in vitro metric for the ith drug. As each HR estimate was accompanied by a 95% CI, weighted least squares was used to incorporate the varying uncertainty across drugs, allowing the regression to account for the differing precision of each HR estimate. A 95% confidence band for the fitted regression line was constructed based on the SE of the mean predicted HR values:


$$CI_{95\%}(x)=\hat{HR}(x)\pm t_{0.975,df}\cdot SE_{\hat{HR}(x)}$$

In this equation, $\hat{HR}(x)$ is the predicted HR at a given in vitro metric value x; $t_{0.975,df}$ is the 2-tailed critical value from the Student t distribution at a 95% confidence level, with df representing the df; and $SE_{\hat{HR}(x)}$ is the SE of the estimated mean HR at x, derived from the variance-covariance matrix of the weighted least squares model. The upper and lower confidence limits were visualized as a shaded band around the fitted trend line for each metric. All analyses were performed using SAS (version 9.4; SAS Institute) and Python (version 3.12.12; Python Software Foundation).

### Sensitivity Analysis

A sensitivity analysis was conducted in which the outcome definition was modified by removing the requirement for anticholinergic prescriptions within 60 days of initial DIP diagnosis, allowing for the assessment of potential misclassification. In a second analysis, we recalculated the in vitro metrics using pK_i_ values extracted exclusively from the single-source dataset reported by Sykes et al [14]. Because this recalculation affected only the pK_i_-dependent metrics, the additional evaluation was performed to determine whether the observed in vitro–clinical relationships were robust to variability in pK_i_ data sources.

### Ethical Considerations

This study was approved by the institutional review boards of Gachon University Gil Hospital (1044396-202312-HR-230-01) and Seoul National University Hospital (E-2409-042-1569). The requirement for informed consent was waived by both review boards because the study involved a retrospective analysis of fully anonymized data, and no identifiable personal information was accessed. All procedures adhered to applicable local and national regulations regarding the protection of personal information, privacy, and confidentiality. As this study involved only secondary analysis of existing anonymized data, no compensation was provided to participants. All personal information was encrypted to ensure that the individuals could not be identified, and access to the dataset was restricted to authorized investigators in accordance with institutional data governance policies.

## Results

### In Vitro DIP Risks

**Figure 2.**
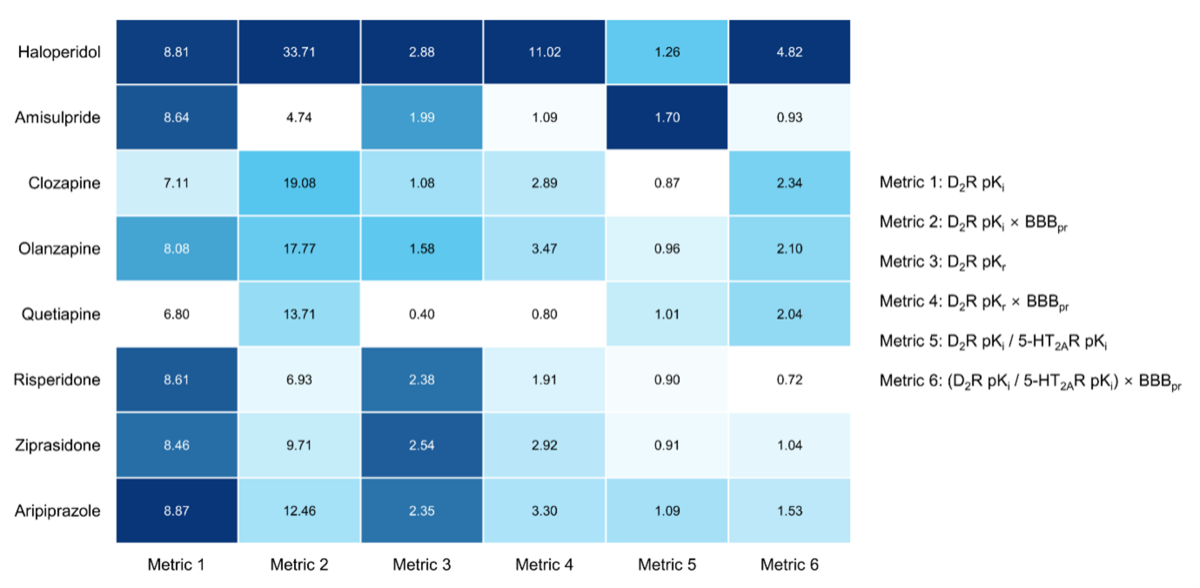
Calculated in vitro risk metrics for drug-induced parkinsonism. The intensity of the blue color represents the relative magnitude of the antipsychotic drug’s metric within the same in vitro measurement. 5-HT_2A_R: serotonin 2A receptor; BBB_pr_: blood-brain barrier penetration rate; D_2_R: dopamine D2 receptor.

The in vitro pharmacological characteristics of APDs used to derive the 6 in vitro DIP risk metrics are summarized in Table S3 in Multimedia Appendix 1, and corresponding metric values are visualized in Figure 2. Haloperidol showed consistently high values across metrics, ranking first in 4 of the 6 metrics. In contrast, quetiapine exhibited the lowest overall metric values. Amisulpride ranked lower on most measures but held first place in metric 5 (D_2_R pK_i_/5-HT_2A_R pK_i_ = 1.70), reflecting its particularly low affinity for 5-HT_2A_R. Clozapine showed a mixed pattern: although it had relatively low D_2_R pK_r_, its BBB penetration rate–adjusted values were higher, placing it second in both metric 2 (D_2_R pK_i_ × BBB penetration rate = 19.08) and metric 6 (D_2_R pK_i_/5-HT_2A_R pK_i_ × BBB penetration rate = 2.34). Olanzapine, risperidone, and ziprasidone generally exhibited low to intermediate levels across metrics. Aripiprazole showed high values for several metrics, ranking first in metric 3 (D_2_R pK_i_=8.87) despite its distinct partial agonist mechanism.

### Patient Characteristics

**Figure 3.**
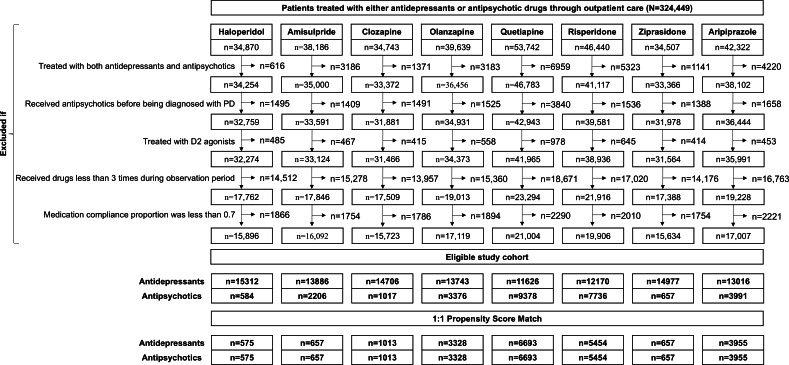
Flowchart of real-world data analysis. PD: Parkinson disease.

A total of 324,449 patients who were exclusively prescribed APDs or SSRIs during outpatient visits were included in the cohort (Figure 3). After eligibility assessment, the final eligible cohort included 109,436 SSRI users and 28,945 APD users. Table 1 presents the baseline characteristics of the haloperidol cohort as a representative example, and those of the remaining cohorts are shown in Tables S4-S10 in Multimedia Appendix 1. Before matching, SSRI users were generally older, whereas APD users showed higher anticonvulsant use. After 1:1 propensity score matching, the final matched cohort included the following numbers for each drug: 575 for haloperidol, 657 for amisulpride, 1013 for clozapine, 3328 for olanzapine, 6693 for quetiapine, 5454 for risperidone, 657 for ziprasidone, and 3955 for aripiprazole. Differences in age and anticonvulsant use, along with other minor discrepancies, were substantially reduced, resulting in well-balanced matched cohorts. Standardized differences for all covariates were below 0.1 across all study cohorts. The median follow-up period was 287 (IQR 142‐366) days across all participants, with a median of 298 (IQR 159‐366) days in the SSRI group and 272 (IQR 131‐364) days in the APD group. The median time to DIP was 71 (IQR 28‐171) days for the entire dataset, 90 (IQR 39‐194) days for the SSRI group, and 63 (IQR 23‐157) days for the APD group.

**Table 1.** Baseline characteristics of the haloperidol cohort.

Variable	Before matching			After matching		
	SSRI^a^ (n=15,312)	Haloperidol (n=584)	Standardized difference	SSRI (n=575)	Haloperidol (n=575)	Standardized difference
Sex (male), n (%)	6789 (44.3)	362 (62.0)	0.3	361 (62.8)	356 (61.9)	−0.01
Age (y), mean (SD)	44.0 (20.7)	39.0 (22.7)	−0.2	41.4 (22.4)	38.6 (22.6)	−0.1
Comorbidities, n (%)						
COPD^b^	76 (0.5)	6 (1.0)	0.06	5 (0.9)	5 (0.9)	0
Dementia	1074 (7.0)	21 (3.6)	−0.1	18 (3.1)	21 (3.7)	0.02
DM^c^	695 (4.5)	38 (6.5)	0.08	33 (5.7)	33 (5.7)	0
Dyslipidemia	725 (4.7)	17 (2.9)	−0.09	21 (3.7)	17 (3.0)	−0.03
ESRD^d^	53 (0.3)	9 (1.5)	0.1	5 (0.9)	8 (1.4)	0.04
Gout	25 (0.2)	2 (0.3)	0.03	1 (0.2)	2 (0.3)	0.03
Hypertension	978 (6.4)	27 (4.6)	−0.07	27 (4.7)	26 (4.5)	−0.008
Liver disease	7 (0.0)	12 (2.1)	0.2	4 (0.7)	3 (0.5)	−0.02
Osteoarthritis	329 (2.1)	6 (1.0)	−0.09	4 (0.7)	6 (1.0)	0.03
Osteoporosis	197 (1.3)	10 (1.7)	0.03	8 (1.4)	9 (1.6)	0.01
Stroke	1412 (9.2)	47 (8.0)	−0.04	50 (8.7)	45 (7.8)	−0.03
Concurrent medications, n (%)						
ACEIs^e^ and ARBs^f^	1223 (8.0)	52 (8.9)	0.03	53 (9.2)	50 (8.7)	−0.01
BBs^g^	2545 (16.6)	136 (23.3)	0.1	135 (23.5)	129 (22.4)	−0.02
CCBs^h^	1269 (8.3)	74 (12.7)	0.1	68 (11.8)	67 (11.7)	−0.005
Anticonvulsants	2084 (13.6)	138 (23.6)	0.2	153 (26.6)	138 (24.0)	−0.06
Anxiolytics	8643 (56.4)	286 (49.0)	−0.1	231 (40.2)	279 (48.5)	0.1
ESAs^i^	74 (0.5)	13 (2.2)	0.1	8 (1.4)	12 (2.1)	0.05
Iron	14 (0.1)	2 (0.3)	0.05	0 (0.0)	2 (0.3)	0.08
Loop diuretics	302 (2.0)	58 (9.9)	0.3	41 (7.1)	50 (8.7)	0.05
Other diuretics	743 (4.9)	72 (12.3)	0.2	57 (9.9)	63 (11.0)	0.03
Statins	1403 (9.2)	54 (9.2)	0.003	52 (9.0)	49 (8.5)	−0.01
AGIs^j^	55 (0.4)	2 (0.3)	−0.003	0 (0.0)	2 (0.3)	0.08
DPP4^k^ inhibitors	179 (1.2)	26 (4.5)	0.2	20 (3.5)	21 (3.7)	0.009
GLP-1^l^ agonists	2 (0.0)	0 (0.0)	−0.01	0 (0.0)	0 (0.0)	0
Insulin	247 (1.6)	41 (7.0)	0.2	31 (5.4)	34 (5.9)	0.02
Meglitinides	28 (0.2)	2 (0.3)	0.03	2 (0.3)	2 (0.3)	0
Metformin	441 (2.9)	34 (5.8)	0.1	29 (5.0)	30 (5.2)	0.007
SGLT2^m^ inhibitors	29 (0.2)	3 (0.5)	0.05	2 (0.3)	3 (0.5)	0.02
Sulfonylurea	291 (1.9)	19 (3.3)	0.08	15 (2.6)	16 (2.8)	0.01

a SSRI: selective serotonin reuptake inhibitor.

b COPD: chronic obstructive pulmonary disease.

c DM: diabetes mellitus.

d ESRD: end-stage renal disease.

e ACEI: angiotensin-converting enzyme inhibitor.

f ARB: angiotensin II receptor blocker.

g BB: beta-blocker.

h CCB: calcium channel blocker.

i ESA: erythropoiesis-stimulating agent.

j AGI: alpha-glucosidase inhibitor.

k DPP4: dipeptidyl peptidase-4.

l GLP-1: glucagonlike peptide-1.

m SGLT2: sodium-glucose cotransporter-2.

### Real-World DIP Risks

The HRs and 95% CIs for DIP across various medications are summarized in Table 2. The typical APD, haloperidol, had the highest DIP risk (HR=4.56, 95% CI 2.29‐9.07). Among the atypical APDs, clozapine (HR=3.59, 95% CI 2.33‐5.52), olanzapine (HR=3.53, 95% CI 2.68‐4.66), risperidone (HR=3.16, 95% CI 2.45‐4.06), and ziprasidone (HR=3.04, 95% CI 1.68‐5.50) showed relatively higher risks. Lower HRs were observed with amisulpride (HR=2.36, 95% CI 1.67‐3.34), quetiapine (HR=2.21, 95% CI 1.76‐2.78), and aripiprazole (HR=2.11, 95% CI 1.56‐2.86). Overall, among the medications analyzed, haloperidol exhibited the highest DIP risk, whereas aripiprazole and quetiapine were associated with the lowest risks.

**Table 2.** Hazard ratios of drug-induced parkinsonism associated with antipsychotic drugs vs selective serotonin reuptake inhibitors.

Antipsychotic drug	Hazard ratio (95% CI)
Haloperidol	4.56 (2.29‐9.07)
Amisulpride	2.36 (1.67‐3.34)
Clozapine	3.59 (2.33‐5.52)
Olanzapine	3.53 (2.68‐4.66)
Quetiapine	2.21 (1.76‐2.78)
Risperidone	3.16 (2.45‐4.06)
Ziprasidone	3.04 (1.68‐5.50)
Aripiprazole	2.11 (1.56‐2.86)

### Correlation Between In Vitro and Real-World DIP Risks

**Figure 4.**
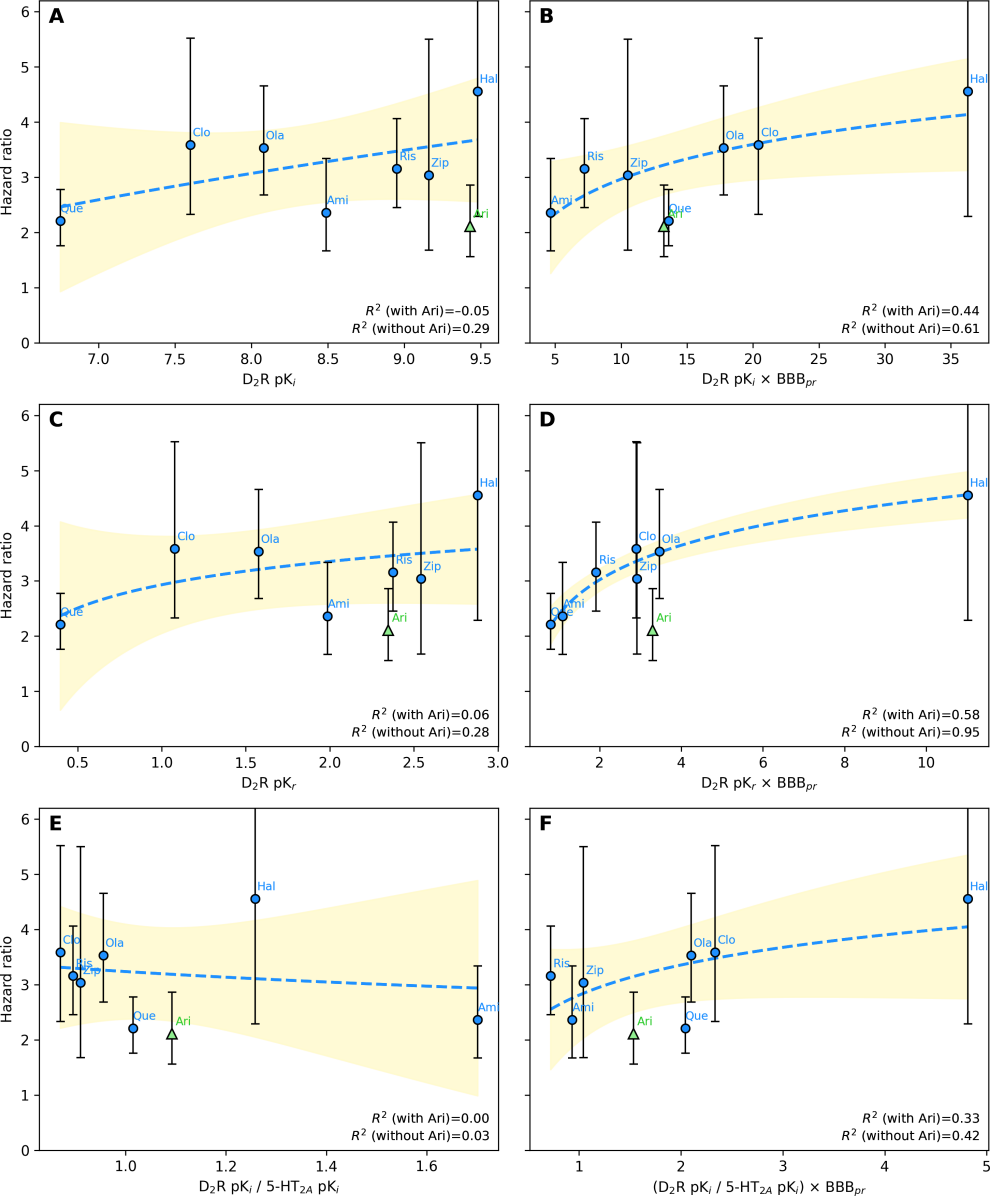
Correlation analysis of in vitro metrics with clinical risk of drug-induced parkinsonism: (A) dopamine D2 receptor (D_2_R) pK_i_ (metric 1); (B) D_2_R pK_i_ × blood-brain barrier penetration rate (BBB_pr_; metric 2); (C) D_2_R pK_r_ (metric 3); (D) D_2_R pK_r_ × BBB_pr_ (metric 4); (E) D_2_R pK_i_/serotonin 2A receptor (5-HT_2A_R) pK_i_ (metric 5); and (F) D_2_R pK_i_/5-HT_2A_R pK_i_ × BBB_pr_ (metric 6). The vertical error bars represent the 95% CIs of the hazard ratios. The blue dashed line and yellow-shaded region indicate the logarithmic regression and 95% confidence band, respectively. The coefficient of determination (*R*^2^) is shown for models calculated with and without aripiprazole (Ari). Ami: amisulpride; Clo: clozapine; Hal: haloperidol; Ola: olanzapine; Ris: risperidone; Zip: ziprasidone.

**Figure 5.**
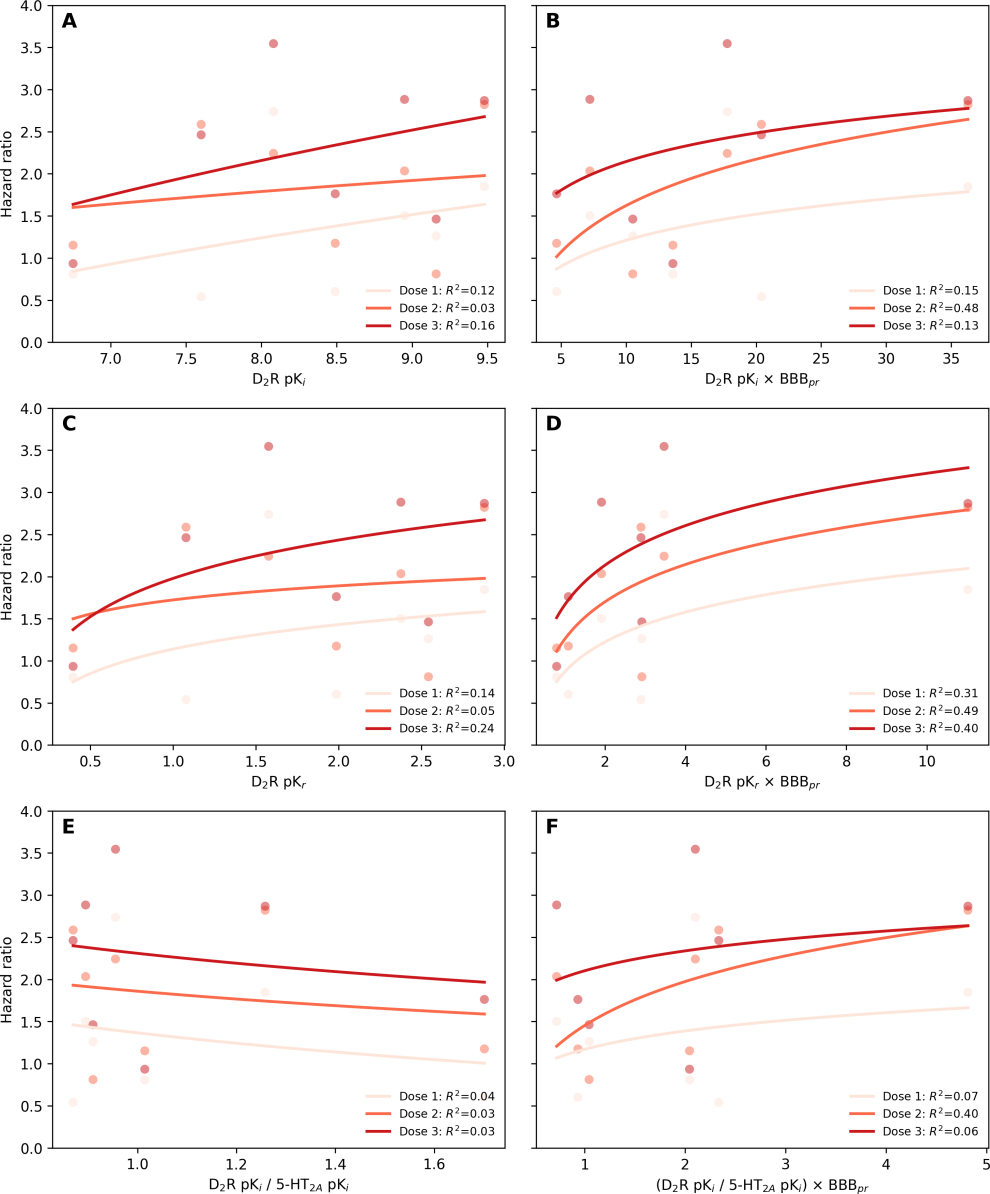
Dose-response analysis of in vitro metrics with clinical risk of drug-induced parkinsonism: (A) dopamine D2 receptor (D_2_R) pK_i_ (metric 1); (B) D_2_R pK_i_ × blood-brain barrier penetration rate (BBB_pr_; metric 2); (C) D_2_R pK_r_ (metric 3); (D) D_2_R pK_r_ × BBB_pr_ (metric 4); (E) D_2_R pK_i_/serotonin 2A receptor (5-HT_2A_R) pK_i_ (metric 5); and (F) D_2_R pK_i_/5-HT_2A_R pK_i_ × BBB_pr_ (metric 6). Each curve represents the fitted logarithmic regression within a dose stratum. Dose 1 (<0.5 the defined daily dose [DDD]), dose 2 (0.5‐1.5 the DDD), and dose 3 (≥1.5 the DDD) are shown in light, intermediate, and dark red, respectively. The coefficient of determination (*R*^2^) is shown for models calculated without aripiprazole.

Among the 6 in vitro metrics evaluated, metric 4 (D_2_R pK_r_ × BBB penetration rate) demonstrated the strongest correlation with real-world DIP risk (*R*^2^=0.95), whereas metric 5 (D_2_R pK_i_/5-HT_2A_R pK_i_) showed the weakest correlation (*R*^2^=0.03; Figure 4). Incorporating BBB penetration markedly improved the explanatory strength across all metric pairs (metric 1 vs metric 2, metric 3 vs metric 4, and metric 5 vs metric 6). Aripiprazole, a partial D_2_R agonist, deviated from the pattern observed among D_2_R antagonists. When aripiprazole was included in the analysis for metric 4, the correlation coefficient decreased substantially from 0.95 to 0.58, reflecting its fundamentally different pharmacological mechanism. Across all 6 metrics, stratification by DDD suggested a trend toward higher DIP risk with increasing exposure (Figure 5). For metric 4, the stratified curves showed the clearest parallel pattern across exposure groups, although the explanatory strength declined within strata.

### Sensitivity Analysis

In the sensitivity analysis using an outcome definition without anticholinergic confirmation, all overall correlation patterns were preserved, and metric 4 continued to show the strongest correlation, with *R*^2^=0.83 (Figure S1 in Multimedia Appendix 1). When the in vitro metrics were recalculated using pK_i_ values derived from a single-study source, the overall correlation pattern remained similar (Table S11 in Multimedia Appendix 1).

## Discussion

### Principal Findings

In this study, we demonstrated that in vitro–derived DIP risk was closely related to the risk of DIP due to the use of APDs observed in real-world clinical settings. Previous studies have evaluated the inhibitory effects of APDs on D_2_Rs or 5-HT_2A_R through in vitro experiments [14,36-38]. However, such in vitro receptor affinity measures alone often fail to reliably predict the frequency of clinical adverse events such as DIP [24,25]. Conversely, RWD studies have assessed the impact of APDs on the incidence of DIP. However, most of these studies have often been limited by the evaluation of only a small subset of medications or lack of appropriate active comparator groups [30,39-43]. Consequently, numerous randomized controlled trials comparing the risk of DIP among patients taking APDs have been conducted over time, resulting in significant expenditure of time and resources [44-51]. A notable strength of this study is that it provides a comprehensive comparison of DIP risks associated with 8 commonly used APDs within a single institutional cohort using robust matching techniques to minimize confounding and demonstrating the relationship between in vitro pharmacological metrics and observed clinical outcomes. These findings suggest that integrating in vitro pharmacological data with clinical evidence may help generate hypotheses for future safety evaluation frameworks, particularly in early exploratory stages of drug development.

Consistent with previous literature, APDs significantly increased the risk of DIP compared to SSRIs, which served as active comparators [14,40,52]. Haloperidol, a typical APD, exhibited the highest DIP risks in both the in vitro metrics and the real-world analysis, aligning with its strong D_2_R antagonist properties. In contrast, atypical APDs showed lower HRs, with aripiprazole exhibiting the lowest DIP risk despite its relatively high receptor affinity and kinetic parameters (pK_i_, pK_r_, and pK_r_ × BBB penetration rate). Sensitivity analyses supported the robustness of the overall associations: using an outcome definition without anticholinergic confirmation yielded correlation patterns that were directionally similar, and recalculating the in vitro metrics with pK_i_ values derived from a single-study source produced a correlation structure comparable to that of the main analysis. Although the DDD-based stratification was exploratory, the generally increasing DIP risk across exposure levels offers qualitative support for a dose-response pattern consistent with prior pharmacological understanding of D_2_R blockade [18,40]. This pattern was most apparent for metric 4, which showed a broadly similar ranking across dose strata. However, the reduced explanatory strength within strata suggests that dose-based subgrouping introduces analytic instability, likely reflecting loss of covariate balance and reduced variability after stratification. These observations indicate that, while dose may influence the in vitro–clinical relationship, larger datasets with broader dosing distributions and more detailed exposure metrics, such as treatment duration and receptor occupancy, will be required to more robustly characterize dose-dependent effects.

Aripiprazole deviated from the general correlation pattern, presenting a lower HR than that expected from metric 4 (D_2_R pK_r_ × BBB penetration rate), with similar discrepancies across other metrics. This divergence is most likely attributable to the unique pharmacodynamic profile of aripiprazole as a partial D_2_R agonist [35]. Unlike full antagonists, aripiprazole displays intrinsic activity at the D_2_Rs, enabling it to maintain a degree of dopaminergic signaling even at high receptor occupancy. A number of positron emission tomography studies have demonstrated that therapeutic doses of aripiprazole achieve high D_2_R occupancy without producing corresponding extrapyramidal adverse effects, indicating that occupancy does not translate directly into functional blockade for partial agonists [53-55]. Additionally, aripiprazole exhibits functional selectivity and stabilizes D_2_R signaling rather than fully suppressing it, which reduces the likelihood of inducing movement disorders despite strong binding [35]. Such pharmacological nuances highlight that future extensions of this framework may require incorporating measures of functional efficacy, such as intrinsic activity, partial agonist efficacy, or occupancy-response weighting, to more accurately model APDs whose clinical effects diverge from antagonist-based predictions.

Another key finding of this study was that K_r_ better reflects the DIP risks observed in clinical settings than K_i_. This observation aligns with that made in a previous study by Sykes et al [14], which highlighted the importance of rebinding kinetics in DIP. Our study extends these findings by integrating BBB penetration rate, which improved the strength of the correlation across all metrics. Among the models tested, the combination of D_2_R pK_r_ and BBB penetration rate (metric 4) showed the strongest correlation with clinical DIP risk. Notably, the correlation coefficient increased from 0.28 (pK_r_ alone) to 0.95 (pK_r_ × BBB penetration rate), indicating that accounting for CNS exposure meaningfully shifted the strength of the association. Conversely, the metrics that incorporated D_2_R K_i_ did not sufficiently reflect the variations in real-world DIP risk. Taken together, these findings suggest that CNS accessibility and rebinding kinetics capture important mechanistic features underlying observed differences among APDs.

Several studies have illustrated the relevance of incorporating BBB penetration rate into risk models. Amisulpride and risperidone, both of which have BBB penetration rate values below 1 (0.55 and 0.81, respectively), appeared below the trend line in unadjusted models, indicating a lower-than-expected DIP risk despite high in vitro receptor affinity (Figures 4A and 4C). This discrepancy is likely due to the limited BBB penetration. When BBB penetration rate–adjusted metrics were applied (Figures 4B and 4D), both drugs aligned closer to the trend line. A similar trend was observed for olanzapine and clozapine, which showed comparable HRs in the clinical data. Although olanzapine exhibited a 46% higher pK_r_ value than clozapine, this difference was reduced to 20% after adjusting for BBB penetration rate, suggesting that the superior BBB penetration of clozapine may compensate for its lower binding affinity, highlighting the importance of accounting for BBB permeability in DIP risk prediction.

### Limitations

Our study had several limitations. First, the in vitro indicators presented herein are aggregate estimates that are not intended for individual patient prediction. Future research could develop individualized risk prediction models that incorporate these indicators. Second, because most patients in the cohort were treated with relatively low APD doses (≤1.0 the DDD), dose-response characterization was inherently limited, and the relationship between the in vitro metrics and clinical risk across the full dosing spectrum remains unvalidated. Future studies encompassing a wider dosing distribution will be needed to more fully delineate dose-dependent patterns. Third, while the metrics demonstrated correlation with real-world DIP risk among D_2_R antagonists, the analysis could not cover all APDs owing to insufficient prescribing frequency, and the current framework does not incorporate intrinsic efficacy, limiting its applicability to partial agonists such as aripiprazole. Therefore, expansion to additional APDs and methodological extensions that account for intrinsic efficacy will be essential for assessing generalizability. Finally, this study used a single-institution CDM database; prescribing patterns and clinical characteristics outside this setting were not captured, and residual confounding inherent to the observational nature may remain despite rigorous propensity score matching. In addition, the identification of DIP onset, even with an enhanced operational definition, may still allow for potential misclassification. Multicenter studies will be valuable to further evaluate the generalizability of the proposed framework. Given these constraints, the findings should be interpreted as exploratory associations rather than definitive causal inferences.

### Conclusions

In conclusion, this study demonstrated that combining receptor-binding kinetics with BBB penetration provides robust in vitro metrics that strongly correlate with the real-world clinical risk of DIP. These findings underscore the importance of integrating receptor kinetics and neuropharmacokinetics with real-world evidence, offering a conceptual foundation that may support more mechanistically informed approaches to future pharmacovigilance and adverse event predictions. Ultimately, such integrative methodologies may help refine early safety assessment and improve decision-making across the drug development continuum, potentially reducing the cost and duration of clinical trials.

## Supplementary material

10.2196/81876 Multimedia Appendix 1 Tables S1-S11 and Figure S1.

10.2196/81876 Checklist 1 STROBE checklist.

## Abbreviations

5-HT_2A_R: serotonin 2A receptor
APD: antipsychotic drug
BBB: blood-brain barrier
CDM: common data model
CNS: central nervous system
D_2_R: dopamine D2 receptor
DDD: defined daily dose
DIP: drug-induced parkinsonism
HR: hazard ratio
RWD: real-world data
SSRI: selective serotonin reuptake inhibitor
STROBE: Strengthening the Reporting of Observational Studies in Epidemiology
