# Supplementary material for: Comparison of In Vitro Metrics With Real-World Risk of Drug-Induced Parkinsonism Due to Antipsychotic Drugs: Retrospective Cohort Study
Source: JMIR Public Health Surveill. 2026 Jan 28;12:e81876. doi: 10.2196/81876 (PMC12850050; doi:10.2196/81876)
Supplement: Multimedia Appendix 1 [file publichealth-v12-e81876-s001.DOC]

**Comparison of In Vitro Metrics with Real-World Risk of Drug-Induced Parkinsonism: Evaluation of Antipsychotic Drugs**

**SUPPLEMENTARY INFORMATION**

Table S1. List of ICD-10 codes for comorbidities

Table S2. List of WHO-ATC codes for concurrent medications

Table S3. In vitro pharmacological characteristics of antipsychotic drugs included in the study

Table S4. Baseline characteristic for amisulpride

Table S5. Baseline characteristic for clozapine

Table S6. Baseline characteristic for olanzapine

Table S7. Baseline characteristic for quetiapine

Table S8. Baseline characteristic for risperidone

Table S9. Baseline characteristic for ziprasidone

Table S10. Baseline characteristic for aripiprazole

Table S11. Sensitivity analysis of single source inhibition constant (K_i_)

Figure S1. Sensitivity analysis of in vitro metrics with clinical risk of drug-induced parkinsonism

**Table S1.** List of ICD-10 codes for comorbidities

| **Comorbidities** | **ICD-10 Code** |
| --- | --- |
| COPD | J41–J44, J47 |
| Dementia | F00–F03, G30 |
| DM | E10–E14 |
| Dyslipidemia | E78.* |
| ESRD | N18.5, N18.6, Z99.2 |
| Gout | M10.* |
| Hypertension | I10–I15 |
| Liver disease | K70–K77 |
| Osteoarthritis | M15–M19 |
| Osteoporosis | M80–M82 |
| Stroke | I60–I64, I69.* |

COPD, chronic obstructive pulmonary disease; DM, diabetes mellitus; ESRD, end stage renal disease; ICD-10, International Classification of Disease 10th Revision

**Table S2.** List of WHO-ATC codes for concurrent medications

| **Class** | **WHO-ATC codes** |
| --- | --- |
| ACEI & ARB | C09A*, C09B*, C09C*, C09D* |
| BB | C07* |
| CCB | C08* |
| Anticonvulsant | N03A* |
| Anxiolytic | N05B* |
| ESA | B03XA01, B03XA02, B03XA03 |
| Iron | B03AA*, B03AB*, B03AC* |
| Loop diuretics | C03C* |
| Other diuretics | C03A*, C03B*, C03D*, C03E* |
| Statin | C10AA*, C10BA* |
| AGIs | A10BF* |
| DPP4 inhibitors | A10BH* |
| GLP-1 agonists | A10BJ* |
| Insulin | A10A* |
| Meglitinides | A10BX02, A10BX03, A10BX04 |
| Metformin | A10BA02, A10BD02, A10BD03, A10BD05, A10BD07, A10BD08, A10BD10, A10BD11, A10BD13, A10BD14, A10BD15, A10BD16, A10BD17, A10BD18, A10BD20, A10BD22, A10BD23, A10BD25, A10BD26, A10BD27, A10BD28 |
| SGLT2 inhibitors | A10BD15, A10BD16, A10BD19, A10BD20, A10BD21, A10BD23, A10BD24, A10BD25, A10BD27, A10BD29, A10BD30; A10BK* |
| Sulfonylurea | A10BB*; A10BD01, A10BD02, A10BD04, A10BD06 |

ACEI, angiotensin-converting enzyme inhibitor; AGIs, α-glucosidase Inhibitors; ARB, angiotensin II receptor blocker; ATC, Anatomical Therapeutic Chemical; BB, beta blocker; CCB, calcium channel blocker; DPP4 inhibitors, dipeptidyl peptidase 4 inhibitors; ESA, erythropoiesis-stimulating agents; GLP-1 agonists, glucagon-like peptide 1 agonists; SGLT2 inhibitors, sodium-glucose cotransporter-2 inhibitors; WHO, World Health Organization.

**Table S3.** In vitro pharmacological characteristics of antipsychotic drugs included in the study

| **Antipsychotic drugs** | **5-HT_2A_R pK_i_** | **D_2_R pK_i_** | **D_2_R pK_r_** | **BBB_pr_** |
| --- | --- | --- | --- | --- |
| Haloperidol | 7.00 | 8.81 | 2.88 | 3.83 |
| Amisulpride | 5.08 | 8.64 | 1.99 | 0.55 |
| Clozapine | 8.17 | 7.11 | 1.08 | 2.68 |
| Olanzapine | 8.45 | 8.08 | 1.58 | 2.20 |
| Quetiapine | 6.71 | 6.80 | 0.40 | 2.01 |
| Risperidone | 9.61 | 8.61 | 2.38 | 0.81 |
| Ziprasidone | 9.30 | 8.46 | 2.54 | 1.15 |
| Aripiprazole | 8.12 | 8.87 | 2.35 | 1.40 |

5-HT_2A_R, serotonin 2A receptor; BBB_pr_, blood brain barrier penetration ratio; D_2_R, dopamine D2 receptor.

**Table S4.** Baseline characteristic for amisulpride cohort

|  | **Pre-Match** | | | **Post-Match** | | |
| --- | --- | --- | --- | --- | --- | --- |
| **Variables** | **SSRI**  **(N=13,886)** | **Amisulpride**  **(N=2206)** | **STD** | **SSRI**  **(N=2205)** | **Amisulpride**  **(N=2205)** | **STD** |
| Sex (male), n (%) | 6238 (44.9) | 845 (38.3) | -0.1 | 846 (38.4) | 844 (38.3) | -0.001 |
| Age (year), mean ± SD | 43.6 ± 21.0 | 50.1 ± 18.1 | 0.3 | 49.2 ± 18.5 | 50.1 ± 18.1 | 0.04 |
| Comorbidities, n (%) | | | | | | |
| COPD | 71 (0.5) | 14 (0.6) | 0.01 | 16 (0.7) | 14 (0.6) | -0.01 |
| Dementia | 1024 (7.4) | 92 (4.2) | -0.1 | 76 (3.5) | 92 (4.2) | 0.03 |
| DM | 615 (4.4) | 134 (6.1) | 0.07 | 107 (4.9) | 133 (6) | 0.05 |
| Dyslipidemia | 662 (4.8) | 119 (5.4) | 0.02 | 94 (4.3) | 119 (5.4) | 0.05 |
| ESRD | 53 (0.4) | 7 (0.3) | -0.01 | 7 (0.3) | 7 (0.3) | 0 |
| Gout | 27 (0.2) | 1 (0.05) | -0.04 | 0 (0) | 1 (0.05) | 0.03 |
| Hypertension | 867 (6.2) | 167 (7.6) | 0.05 | 154 (7) | 166 (7.5) | 0.02 |
| Liver disease | 5 (0.04) | 5 (0.23) | 0.05 | 3 (0.14) | 4 (0.18) | 0.01 |
| Osteoarthritis | 285 (2.1) | 52 (2.4) | 0.02 | 43 (2) | 52 (2.4) | 0.02 |
| Osteoporosis | 161 (1.2) | 40 (1.8) | 0.05 | 22 (1) | 40 (1.8) | 0.06 |
| Stroke | 1289 (9.3) | 203 (9.2) | -0.003 | 186 (8.4) | 203 (9.2) | 0.02 |
| Concurrent medications, n (%) | | | | | | |
| ACEI&ARB | 1100 (7.9) | 190 (8.6) | 0.02 | 174 (7.9) | 189 (8.6) | 0.02 |
| BB | 2218 (16) | 508 (23) | 0.1 | 532 (24.1) | 508 (23) | -0.02 |
| CCB | 1126 (8.1) | 270 (12.2) | 0.1 | 246 (11.2) | 270 (12.2) | 0.03 |
| Anticonvulsant | 1785 (12.9) | 412 (18.7) | 0.1 | 470 (21.3) | 412 (18.7) | -0.06 |
| Anxiolytic | 7508 (54.1) | 1733 (78.6) | 0.5 | 1714 (77.7) | 1732 (78.6) | 0.02 |
| ESA | 69 (0.5) | 16 (0.7) | 0.02 | 19 (0.9) | 16 (0.7) | -0.01 |
| Iron | 12 (0.1) | 2 (0.1) | 0.001 | 0 (0) | 2 (0.1) | 0.04 |
| Loop diuretics | 264 (1.9) | 94 (4.3) | 0.1 | 92 (4.2) | 93 (4.2) | 0.002 |
| Other diuretics | 656 (4.7) | 153 (6.9) | 0.09 | 144 (6.5) | 152 (6.9) | 0.01 |
| Statin | 1265 (9.1) | 246 (11.2) | 0.06 | 208 (9.4) | 246 (11.2) | 0.05 |
| AGIs | 45 (0.3) | 11 (0.5) | 0.02 | 8 (0.4) | 11 (0.5) | 0.02 |
| DPP4 inhibitors | 172 (1.2) | 30 (1.4) | 0.01 | 21 (1) | 29 (1.3) | 0.03 |
| GLP-1 agonists | 1 (0.01) | 0 (0) | -0.01 | 0 (0) | 0 (0) | 0 |
| Insulin | 215 (1.6) | 68 (3.1) | 0.1 | 53 (2.4) | 67 (3) | 0.03 |
| Meglitinides | 24 (0.2) | 6 (0.3) | 0.02 | 4 (0.2) | 6 (0.3) | 0.01 |
| Metformin | 398 (2.9) | 87 (3.9) | 0.05 | 67 (3) | 86 (3.9) | 0.04 |
| SGLT2 inhibitors | 30 (0.2) | 0 (0) | -0.06 | 0 (0) | 0 (0) | 0 |
| Sulfonylurea | 256 (1.8) | 67 (3) | 0.07 | 58 (2.6) | 66 (3) | 0.02 |

ACEI, angiotensin-converting enzyme inhibitor; AGIs, α-glucosidase Inhibitors; ARB, angiotensin II receptor blocker; BB, beta blocker; CCB, calcium channel blocker; COPD, chronic obstructive pulmonary disease; DM, diabetes mellitus; DPP4 inhibitors, dipeptidyl peptidase 4 inhibitors; ESA, erythropoiesis-stimulating agents; ESRD, end stage renal disease; GLP-1 agonists, glucagon-like peptide 1 agonists; SGLT2 inhibitors, sodium-glucose cotransporter-2 inhibitors.

**Table S5.** Baseline characteristic for clozapine cohort

|  | **Pre-Match** | | | **Post-Match** | | |
| --- | --- | --- | --- | --- | --- | --- |
| **Variables** | **SSRI**  **(N=14,706)** | **Clozapine**  **(N=1017)** | **STD** | **SSRI**  **(N=1013)** | **Clozapine**  **(N=1013)** | **STD** |
| Sex (male), n (%) | 6428 (43.7) | 513 (50.4) | 0.1 | 506 (50.0) | 512 (50.5) | 0.01 |
| Age (year), mean ± SD | 44.6 ± 21.0 | 33.6 ± 12.7 | -0.6 | 33.8 ± 13.5 | 33.6 ± 12.7 | -0.01 |
| Comorbidities, n (%) | | | | | | |
| COPD | 77 (0.5) | 0 (0) | -0.1 | 0 (0) | 0 (0) | 0 |
| Dementia | 1076 (7.3) | 8 (0.8) | -0.3 | 7 (0.7) | 8 (0.8) | 0.01 |
| DM | 685 (4.7) | 24 (2.4) | -0.1 | 11 (1.1) | 21 (2.1) | 0.07 |
| Dyslipidemia | 720 (4.9) | 15 (1.5) | -0.2 | 7 (0.7) | 15 (1.5) | 0.07 |
| ESRD | 55 (0.4) | 2 (0.2) | -0.03 | 0 (0) | 2 (0.2) | 0.06 |
| Gout | 26 (0.2) | 2 (0.2) | 0.005 | 1 (0.1) | 2 (0.2) | 0.02 |
| Hypertension | 983 (6.7) | 10 (1) | -0.3 | 4 (0.4) | 10 (1) | 0.07 |
| Liver disease | 7 (0.1) | 1 (0.1) | 0.01 | 0 (0) | 1 (0.1) | 0.04 |
| Osteoarthritis | 329 (2.2) | 1 (0.1) | -0.2 | 0 (0) | 1 (0.1) | 0.04 |
| Osteoporosis | 197 (1.3) | 1 (0.1) | -0.1 | 0 (0) | 1 (0.1) | 0.04 |
| Stroke | 1407 (9.6) | 19 (1.9) | -0.3 | 15 (1.5) | 18 (1.8) | 0.02 |
| Concurrent medications, n (%) | | | | | | |
| ACEI&ARB | 1234 (8.4) | 19 (1.9) | -0.3 | 10 (1) | 19 (1.9) | 0.02 |
| BB | 2353 (16) | 392 (38.5) | 0.5 | 393 (38.8) | 388 (38.3) | 0.02 |
| CCB | 1269 (8.6) | 23 (2.3) | -0.2 | 19 (1.9) | 23 (2.3) | 0.03 |
| Anticonvulsant | 1883 (12.8) | 346 (34) | 0.5 | 325 (32.1) | 343 (33.9) | 0.008 |
| Anxiolytic | 8216 (55.9) | 637 (62.6) | 0.1 | 629 (62.1) | 633 (62.5) | -0.01 |
| ESA | 75 (0.5) | 2 (0.2) | -0.05 | 0 (0) | 2 (0.2) | 0 |
| Iron | 14 (0.1) | 1 (0.1) | 0.001 | 0 (0) | 1 (0.1) | 0.02 |
| Loop diuretics | 309 (2.1) | 3 (0.3) | -0.1 | 2 (0.2) | 3 (0.3) | 0 |
| Other diuretics | 752 (5.1) | 7 (0.7) | -0.2 | 4 (0.4) | 7 (0.7) | -0.02 |
| Statin | 1408 (9.6) | 22 (2.2) | -0.3 | 14 (1.4) | 21 (2.1) | 0.07 |
| AGIs | 54 (0.4) | 2 (0.2) | -0.03 | 1 (0.1) | 2 (0.2) | 0.03 |
| DPP4 inhibitors | 183 (1.2) | 4 (0.4) | -0.09 | 2 (0.2) | 4 (0.4) | 0.06 |
| GLP-1 agonists | 1 (0.01) | 0 (0) | -0.01 | 0 (0) | 0 (0) | 0.01 |
| Insulin | 249 (1.7) | 6 (0.6) | -0.1 | 5 (0.5) | 6 (0.6) | 0.04 |
| Meglitinides | 28 (0.2) | 0 (0) | -0.06 | 0 (0) | 0 (0) | 0.05 |
| Metformin | 432 (2.9) | 29 (2.9) | -0.005 | 17 (1.7) | 25 (2.5) | 0.04 |
| SGLT2 inhibitors | 29 (0.2) | 2 (0.2) | 0 | 2 (0.2) | 1 (0.1) | 0.05 |
| Sulfonylurea | 290 (2) | 5 (0.5) | -0.1 | 1 (0.1) | 5 (0.5) | 0.02 |

ACEI, angiotensin-converting enzyme inhibitor; AGIs, α-glucosidase Inhibitors; ARB, angiotensin II receptor blocker; BB, beta blocker; CCB, calcium channel blocker; COPD, chronic obstructive pulmonary disease; DM, diabetes mellitus; DPP4 inhibitors, dipeptidyl peptidase 4 inhibitors; ESA, erythropoiesis-stimulating agents; ESRD, end stage renal disease; GLP-1 agonists, glucagon-like peptide 1 agonists; SGLT2 inhibitors, sodium-glucose cotransporter-2 inhibitors.

**Table S6.** Baseline characteristic for olanzapine cohort

|  | **Pre-Match** | | | **Post-Match** | | |
| --- | --- | --- | --- | --- | --- | --- |
| **Variables** | **SSRI**  **(N=13,743)** | **Olanzapine**  **(N=3376)** | **STD** | **SSRI**  **(N=3324)** | **Olanzapine**  **(N=3324)** | **STD** |
| Sex (male), n (%) | 6011 (43.7) | 1668 (49.4) | 0.1 | 1583 (47.6) | 1629 (49.0) | 0.02 |
| Age (year), mean ± SD | 44.8 ± 20.9 | 41.4 ± 19.5 | -0.1 | 39.3 ± 18.5 | 41.3 ± 19.5 | 0.1 |
| Comorbidities, n (%) | | | | | | |
| COPD | 67 (0.5) | 33 (1) | 0.05 | 25 (0.8) | 27 (0.8) | 0.006 |
| Dementia | 1029 (7.5) | 168 (5) | -0.1 | 142 (4.3) | 166 (5) | 0.03 |
| DM | 659 (4.8) | 138 (4.1) | -0.03 | 99 (3) | 129 (3.9) | 0.05 |
| Dyslipidemia | 694 (5.1) | 123 (3.6) | -0.06 | 110 (3.3) | 123 (3.7) | 0.02 |
| ESRD | 52 (0.4) | 25 (0.7) | 0.04 | 20 (0.6) | 23 (0.7) | 0.01 |
| Gout | 26 (0.2) | 9 (0.3) | 0.01 | 9 (0.3) | 8 (0.2) | -0.006 |
| Hypertension | 937 (6.8) | 159 (4.7) | -0.09 | 125 (3.8) | 159 (4.8) | 0.05 |
| Liver disease | 7 (0.1) | 11 (0.3) | 0.06 | 6 (0.2) | 7 (0.2) | 0.006 |
| Osteoarthritis | 308 (2.2) | 65 (1.9) | -0.02 | 51 (1.5) | 63 (1.9) | 0.02 |
| Osteoporosis | 189 (1.4) | 34 (1) | -0.03 | 25 (0.8) | 34 (1) | 0.02 |
| Stroke | 1349 (9.8) | 226 (6.7) | -0.11 | 199 (6) | 219 (6.6) | 0.02 |
| Concurrent medications, n (%) | | | | | | |
| ACEI&ARB | 1171 (8.5) | 252 (7.5) | -0.03 | 25 (0.8) | 34 (1) | 0.02 |
| BB | 2114 (15.4) | 923 (27.3) | 0.2 | 918 (27.6) | 885 (26.6) | -0.02 |
| CCB | 1208 (8.8) | 390 (11.6) | 0.09 | 300 (9) | 364 (11) | 0.06 |
| Anticonvulsant | 1636 (11.9) | 1253 (37.1) | 0.6 | 138 (24) | 153 (26.6) | -0.009 |
| Anxiolytic | 7549 (54.9) | 2384 (70.6) | 0.3 | 2295 (69) | 2334 (70.2) | 0.02 |
| ESA | 70 (0.5) | 40 (1.2) | 0.07 | 26 (0.8) | 39 (1.2) | 0.04 |
| Iron | 14 (0.1) | 7 (0.2) | 0.02 | 9 (0.3) | 7 (0.2) | -0.01 |
| Loop diuretics | 278 (2) | 207 (6.1) | 0.2 | 160 (4.8) | 179 (5.4) | 0.02 |
| Other diuretics | 698 (5.1) | 280 (8.3) | 0.1 | 223 (6.7) | 250 (7.5) | 0.03 |
| Statin | 1326 (9.7) | 331 (9.8) | 0.005 | 267 (8) | 317 (9.5) | 0.05 |
| AGIs | 51 (0.4) | 5 (0.2) | -0.04 | 199 (6) | 219 (6.6) | -0.02 |
| DPP4 inhibitors | 167 (1.2) | 75 (2.2) | 0.07 | 53 (1.6) | 65 (2) | 0.02 |
| GLP-1 agonists | 2 (0.06) | 1 (0.03) | 0.01 | 2 (0.06) | 1 (0.03) | -0.01 |
| Insulin | 232 (1.7) | 126 (3.7) | 0.1 | 81 (2.4) | 106 (3.2) | 0.04 |
| Meglitinides | 27 (0.2) | 6 (0.2) | -0.004 | 1 (0.03) | 5 (0.2) | 0.04 |
| Metformin | 409 (3) | 118 (3.5) | 0.02 | 89 (2.7) | 106 (3.2) | 0.03 |
| SGLT2 inhibitors | 28 (0.2) | 5 (0.2) | -0.01 | 2 (0.1) | 5 (0.2) | 0.02 |
| Sulfonylurea | 276 (2) | 54 (1.6) | -0.03 | 42 (1.3) | 52 (1.6) | 0.02 |

ACEI, angiotensin-converting enzyme inhibitor; AGIs, α-glucosidase Inhibitors; ARB, angiotensin II receptor blocker; BB, beta blocker; CCB, calcium channel blocker; COPD, chronic obstructive pulmonary disease; DM, diabetes mellitus; DPP4 inhibitors, dipeptidyl peptidase 4 inhibitors; ESA, erythropoiesis-stimulating agents; ESRD, end stage renal disease; GLP-1 agonists, glucagon-like peptide 1 agonists; SGLT2 inhibitors, sodium-glucose cotransporter-2 inhibitors.

**Table S7.** Baseline characteristic for quetiapine cohort

|  | **Pre-Match** | | | **Post-Match** | | |
| --- | --- | --- | --- | --- | --- | --- |
| **Variables** | **SSRI**  **(N=11,626)** | **Quetiapine**  **(N=9378)** | **STD** | **SSRI**  **(N=6693)** | **Quetiapine**  **(N=6693)** | **STD** |
| Sex (male), n (%) | 5248 (45.1) | 3969 (42.3) | -0.05 | 2612 (39.0) | 2793 (41.7) | 0.05 |
| Age (year), mean ± SD | 44.2 ± 21.0 | 48.9 ± 22.0 | 0.2 | 48.2 ± 20.5 | 49.2 ± 21.4 | 0.04 |
| Comorbidities, n (%) | | | | | | |
| COPD | 56 (0.5) | 115 (1.2) | 0.08 | 54 (0.8) | 53 (0.8) | -0.001 |
| Dementia | 785 (6.8) | 1331 (14.2) | 0.2 | 775 (11.6) | 784 (11.7) | 0.004 |
| DM | 517 (4.5) | 731 (7.8) | 0.1 | 370 (5.5) | 393 (5.9) | 0.01 |
| Dyslipidemia | 558 (4.8) | 663 (7.1) | 0.09 | 410 (6.1) | 424 (6.3) | 0.008 |
| ESRD | 32 (0.3) | 109 (1.2) | 0.10 | 30 (0.5) | 44 (0.7) | 0.02 |
| Gout | 21 (0.2) | 41 (0.4) | 0.04 | 17 (0.3) | 19 (0.3) | 0.005 |
| Hypertension | 741 (6.4) | 809 (8.6) | 0.08 | 488 (7.3) | 520 (7.8) | 0.01 |
| Liver disease | 4 (0) | 31 (0.3) | 0.06 | 4 (0.1) | 9 (0.1) | 0.02 |
| Osteoarthritis | 254 (2.2) | 248 (2.6) | 0.03 | 164 (2.5) | 170 (2.5) | 0.005 |
| Osteoporosis | 142 (1.2) | 157 (1.7) | 0.03 | 101 (1.5) | 104 (1.6) | 0.003 |
| Stroke | 1089 (9.4) | 1191 (12.7) | 0.1 | 693 (10.4) | 740 (11.1) | 0.02 |
| Concurrent medications, n (%) | | | | | | |
| ACEI&ARB | 930 (8) | 1104 (11.8) | 0.1 | 602 (9) | 653 (9.8) | 0.02 |
| BB | 1663 (14.3) | 2058 (21.9) | 0.2 | 1208 (18.1) | 1238 (18.5) | 0.01 |
| CCB | 950 (8.2) | 1486 (15.9) | 0.2 | 765 (11.4) | 813 (12.2) | 0.02 |
| Anticonvulsant | 1184 (10.2) | 2952 (31.5) | 0.5 | 1184 (17.7) | 1141 (17.1) | -0.01 |
| Anxiolytic | 6014 (51.7) | 6267 (66.8) | 0.3 | 4280 (64) | 4252 (63.5) | -0.008 |
| ESA | 49 (0.4) | 139 (1.5) | 0.1 | 43 (0.6) | 52 (0.8) | 0.01 |
| Iron | 9 (0.1) | 25 (0.3) | 0.04 | 9 (0.1) | 11 (0.2) | 0.007 |
| Loop diuretics | 207 (1.8) | 674 (7.2) | 0.2 | 205 (3.1) | 228 (3.4) | 0.01 |
| Other diuretics | 547 (4.7) | 970 (10.3) | 0.2 | 390 (5.8) | 438 (6.5) | 0.03 |
| Statin | 1083 (9.3) | 1426 (15.2) | 0.1 | 779 (11.6) | 817 (12.2) | 0.01 |
| AGIs | 39 (0.3) | 38 (0.4) | 0.01 | 27 (0.4) | 28 (0.4) | 0.002 |
| DPP4 inhibitors | 135 (1.2) | 339 (3.6) | 0.1 | 121 (1.8) | 134 (2) | 0.01 |
| GLP-1 agonists | 2 (0) | 5 (0.1) | 0.01 | 2 (0) | 2 (0) | 0 |
| Insulin | 171 (1.5) | 511 (5.5) | 0.2 | 164 (2.5) | 173 (2.6) | 0.008 |
| Meglitinides | 22 (0.2) | 28 (0.3) | 0.02 | 11 (0.2) | 14 (0.2) | 0.01 |
| Metformin | 330 (2.8) | 568 (6.1) | 0.1 | 261 (3.9) | 267 (4) | 0.004 |
| SGLT2 inhibitors | 24 (0.2) | 29 (0.3) | -0.02 | 13 (0.2) | 16 (0.2) | 0.009 |
| Sulfonylurea | 220 (1.9) | 344 (3.7) | 0.1 | 159 (2.4) | 169 (2.5) | 0.009 |

ACEI, angiotensin-converting enzyme inhibitor; AGIs, α-glucosidase Inhibitors; ARB, angiotensin II receptor blocker; BB, beta blocker; CCB, calcium channel blocker; COPD, chronic obstructive pulmonary disease; DM, diabetes mellitus; DPP4 inhibitors, dipeptidyl peptidase 4 inhibitors; ESA, erythropoiesis-stimulating agents; ESRD, end stage renal disease; GLP-1 agonists, glucagon-like peptide 1 agonists; SGLT2 inhibitors, sodium-glucose cotransporter-2 inhibitors.

**Table S8.** Baseline characteristic for risperidone cohort

|  | **Pre-Match** | | | **Post-Match** | | |
| --- | --- | --- | --- | --- | --- | --- |
| **Variables** | **SSRI**  **(N=12,170)** | **Risperidone**  **(N=7736)** | **STD** | **SSRI**  **(N=5454)** | **Risperidone**  **(N=5454)** | **STD** |
| Sex (male), n (%) | 4965 (40.8) | 4783 (61.8) | 0.4 | 2878 (52.8) | 2781 (51.0) | -0.03 |
| Age (year), mean ± SD | 46.7 ± 20.1 | 31.2 ± 23.7 | -0.7 | 37.6 ± 20.4 | 38.5 ± 23.3 | 0.04 |
| Comorbidities, n (%) | | | | | | |
| COPD | 64 (0.5) | 19 (0.3) | -0.04 | 15 (0.3) | 17 (0.3) | 0.006 |
| Dementia | 810 (6.7) | 770 (10) | 0.1 | 612 (11.2) | 682 (12.5) | 0.04 |
| DM | 602 (5) | 252 (3.3) | -0.08 | 190 (3.5) | 213 (3.9) | 0.02 |
| Dyslipidemia | 650 (5.3) | 199 (2.6) | -0.1 | 162 (3) | 183 (3.4) | 0.02 |
| ESRD | 43 (0.4) | 37 (0.5) | 0.01 | 26 (0.5) | 27 (0.5) | 0.002 |
| Gout | 24 (0.2) | 10 (0.13) | -0.01 | 10 (0.2) | 9 (0.2) | -0.004 |
| Hypertension | 883 (7.3) | 280 (3.6) | -0.1 | 231 (4.2) | 242 (4.4) | 0.009 |
| Liver disease | 6 (0.05) | 14 (0.18) | 0.03 | 4 (0.1) | 8 (0.2) | 0.02 |
| Osteoarthritis | 299 (2.5) | 70 (0.9) | -0.1 | 71 (1.3) | 66 (1.2) | -0.008 |
| Osteoporosis | 184 (1.5) | 54 (0.7) | -0.07 | 50 (0.9) | 52 (1) | 0.003 |
| Stroke | 1247 (10.3) | 515 (6.7) | -0.1 | 400 (7.3) | 444 (8.1) | 0.03 |
| Concurrent medications, n (%) | | | | | | |
| ACEI&ARB | 1097 (9) | 390 (5) | -0.1 | 315 (5.8) | 346 (6.3) | 0.02 |
| BB | 1953 (16.1) | 1051 (13.6) | -0.06 | 946 (17.4) | 934 (17.1) | -0.005 |
| CCB | 1134 (9.3) | 494 (6.4) | -0.1 | 399 (7.3) | 430 (7.9) | 0.02 |
| Anticonvulsant | 1397 (11.5) | 2058 (26.6) | 0.3 | 1199 (22) | 1335 (24.5) | 0.05 |
| Anxiolytic | 7137 (58.6) | 2903 (37.5) | -0.4 | 2497 (45.8) | 2665 (48.9) | 0.06 |
| ESA | 56 (0.5) | 56 (0.7) | 0.03 | 39 (0.7) | 40 (0.7) | 0.002 |
| Iron | 13 (0.1) | 6 (0.1) | -0.009 | 3 (0.06) | 4 (0.07) | 0.007 |
| Loop diuretics | 262 (2.2) | 245 (3.2) | 0.06 | 161 (3) | 189 (3.5) | 0.02 |
| Other diuretics | 656 (5.4) | 348 (4.5) | -0.04 | 244 (4.5) | 287 (5.3) | 0.03 |
| Statin | 1274 (10.5) | 457 (5.9) | -0.1 | 779 (11.6) | 370 (6.8) | 0.02 |
| AGIs | 46 (0.4) | 26 (0.3) | -0.007 | 19 (0.4) | 17 (0.3) | -0.006 |
| DPP4 inhibitors | 154 (1.3) | 90 (1.2) | -0.009 | 59 (1.1) | 74 (1.4) | 0.02 |
| GLP-1 agonists | 2 (0.02) | 0 (0) | -0.01 | 1 (0.02) | 0 (0) | -0.01 |
| Insulin | 210 (1.7) | 173 (2.2) | 0.03 | 108 (2) | 131 (2.4) | 0.02 |
| Meglitinides | 24 (0.2) | 11 (0.1) | -0.01 | 12 (0.22) | 11 (0.20) | -0.004 |
| Metformin | 385 (3.2) | 176 (2.3) | -0.05 | 124 (2.3) | 151 (2.8) | 0.03 |
| SGLT2 inhibitors | 26 (0.2) | 8 (0.1) | -0.02 | 13 (0.2) | 6 (0.1) | 0 |
| Sulfonylurea | 247 (2) | 125 (1.6) | -0.03 | 159 (2.4) | 86 (1.6) | 0.01 |

ACEI, angiotensin-converting enzyme inhibitor; AGIs, α-glucosidase Inhibitors; ARB, angiotensin II receptor blocker; BB, beta blocker; CCB, calcium channel blocker; COPD, chronic obstructive pulmonary disease; DM, diabetes mellitus; DPP4 inhibitors, dipeptidyl peptidase 4 inhibitors; ESA, erythropoiesis-stimulating agents; ESRD, end stage renal disease; GLP-1 agonists, glucagon-like peptide 1 agonists; SGLT2 inhibitors, sodium-glucose cotransporter-2 inhibitors.

**Table S9.** Baseline characteristic for ziprasidone cohort

|  | **Pre-Match** | | | **Post-Match** | | |
| --- | --- | --- | --- | --- | --- | --- |
| **Variables** | **SSRI**  **(N=14,997)** | **Ziprasidone**  **(N=657)** | **STD** | **SSRI**  **(N=657)** | **Ziprasidone**  **(N=657)** | **STD** |
| Sex (male), n (%) | 6708 (44.8) | 212 (32.3) | -0.2 | 208 (31.7) | 212 (32.3) | 0.01 |
| Age (year), mean ± SD | 44.3 ± 20.8 | 31.1 ± 11.2 | -0.7 | 30.8 ± 12.6 | 31.1 ± 11.2 | 0.01 |
| Comorbidities, n (%) | | | | | | |
| COPD | 77 (0.5) | 1 (0.2) | -0.06 | 2 (0.3) | 1 (0.2) | -0.03 |
| Dementia | 1078 (7.2) | 6 (0.9) | -0.3 | 3 (0.5) | 6 (0.9) | 0.05 |
| DM | 699 (4.7) | 21 (3.2) | -0.07 | 11 (1.7) | 21 (3.2) | 0.09 |
| Dyslipidemia | 721 (4.8) | 27 (4.1) | -0.03 | 19 (2.9) | 27 (4.1) | 0.06 |
| ESRD | 55 (0.4) | 0 (0) | -0.08 | 0 (0) | 0 (0) | 0 |
| Gout | 26 (0.2) | 1 (0.2) | -0.005 | 1 (0.2) | 1 (0.2) | 0 |
| Hypertension | 983 (6.6) | 12 (1.8) | -0.2 | 18 (2.7) | 12 (1.8) | -0.06 |
| Liver disease | 7 (0.1) | 0 (0) | -0.03 | 0 (0) | 0 (0) | 0 |
| Osteoarthritis | 329 (2.2) | 3 (0.5) | -0.1 | 3 (0.5) | 3 (0.5) | 0 |
| Osteoporosis | 197 (1.3) | 0 (0) | -0.1 | 0 (0) | 0 (0) | 0 |
| Stroke | 1414 (9.4) | 19 (2.9) | -0.2 | 12 (1.8) | 19 (2.9) | 0.07 |
| Concurrent medications, n (%) | | | | | | |
| ACEI&ARB | 1228 (8.2) | 14 (2.1) | -0.2 | 11 (1.7) | 14 (2.1) | 0.03 |
| BB | 2461 (16.4) | 214 (32.6) | 0.3 | 234 (35.6) | 214 (32.6) | -0.06 |
| CCB | 1278 (8.5) | 15 (2.3) | -0.2 | 13 (2) | 15 (2.3) | 0.02 |
| Anticonvulsant | 1940 (13) | 324 (49.3) | 0.8 | 327 (49.8) | 324 (49.3) | -0.009 |
| Anxiolytic | 8383 (56) | 449 (68.3) | 0.2 | 449 (68.3) | 449 (68.3) | 0 |
| ESA | 75 (0.5) | 0 (0) | -0.1 | 0 (0) | 0 (0) | 0 |
| Iron | 14 (0.1) | 1 (0.2) | 0.01 | 1 (0.2) | 1 (0.2) | 0 |
| Loop diuretics | 308 (2.1) | 3 (0.5) | -0.1 | 2 (0.3) | 3 (0.5) | 0.02 |
| Other diuretics | 744 (5) | 12 (1.8) | -0.1 | 9 (1.4) | 12 (1.8) | 0.03 |
| Statin | 1410 (9.4) | 18 (2.7) | -0.2 | 14 (2.1) | 18 (2.7) | 0.04 |
| AGIs | 54 (0.4) | 0 (0) | -0.08 | 0 (0) | 0 (0) | 0 |
| DPP4 inhibitors | 182 (1.2) | 5 (0.8) | -0.04 | 4 (0.6) | 5 (0.8) | 0.01 |
| GLP-1 agonists | 2 (0.01) | 1 (0.2) | 0.04 | 0 (0) | 1 (0.2) | 0.05 |
| Insulin | 249 (1.7) | 3 (0.5) | -0.1 | 4 (0.6) | 3 (0.5) | -0.02 |
| Meglitinides | 27 (0.2) | 0 (0) | -0.06 | 0 (0) | 0 (0) | 0 |
| Metformin | 439 (2.9) | 25 (3.8) | 0.04 | 12 (1.8) | 25 (3.8) | 0.1 |
| SGLT2 inhibitors | 29 (0.2) | 2 (0.3) | 0.02 | 1 (0.2) | 2 (0.3) | 0.03 |
| Sulfonylurea | 292 (2) | 4 (0.6) | -0.1 | 4 (0.6) | 4 (0.6) | 0 |

ACEI, angiotensin-converting enzyme inhibitor; AGIs, α-glucosidase Inhibitors; ARB, angiotensin II receptor blocker; BB, beta blocker; CCB, calcium channel blocker; COPD, chronic obstructive pulmonary disease; DM, diabetes mellitus; DPP4 inhibitors, dipeptidyl peptidase 4 inhibitors; ESA, erythropoiesis-stimulating agents; ESRD, end stage renal disease; GLP-1 agonists, glucagon-like peptide 1 agonists; SGLT2 inhibitors, sodium-glucose cotransporter-2 inhibitors.

**Table S10.** Baseline characteristic for aripiprazole cohort

|  | **Pre-Match** | | | **Post-Match** | | |
| --- | --- | --- | --- | --- | --- | --- |
| **Variables** | **SSRI**  **(N=13,016)** | **Aripiprazole**  **(N=3991)** | **STD** | **SSRI**  **(N=3955)** | **Aripiprazole**  **(N=3955)** | **STD** |
| Sex (male), n (%) | 5840 (44.9) | 1740 (43.6) | -0.02 | 1637 (41.4) | 1733 (43.8) | 0.04 |
| Age (year), mean ± SD | 45.6 ± 21.0 | 35.5 ± 21.4 | -0.4 | 36.4 ± 20.0 | 35.6 ± 21.4 | -0.03 |
| Comorbidities, n (%) | | | | | | |
| COPD | 70 (0.5) | 20 (0.5) | -0.005 | 10 (0.3) | 20 (0.5) | 0.04 |
| Dementia | 1026 (7.9) | 226 (5.7) | -0.08 | 217 (5.5) | 222 (5.6) | 0.005 |
| DM | 631 (4.9) | 158 (4) | -0.04 | 119 (3) | 147 (3.7) | 0.03 |
| Dyslipidemia | 642 (4.9) | 198 (5) | 0.001 | 182 (4.6) | 190 (4.8) | 0.009 |
| ESRD | 47 (0.4) | 15 (0.4) | 0.002 | 11 (0.3) | 14 (0.4) | 0.01 |
| Gout | 25 (0.2) | 8 (0.2) | 0.002 | 2 (0.05) | 8 (0.2) | 0.04 |
| Hypertension | 900 (6.9) | 176 (4.4) | -0.1 | 160 (4.1) | 175 (4.4) | 0.01 |
| Liver disease | 6 (0.1) | 5 (0.1) | 0.02 | 3 (0.08) | 3 (0.08) | 0 |
| Osteoarthritis | 293 (2.3) | 75 (1.9) | -0.02 | 144 (3.6) | 74 (1.9) | -0.1 |
| Osteoporosis | 178 (1.4) | 52 (1.3) | -0.006 | 48 (1.2) | 50 (1.3) | 0.004 |
| Stroke | 1306 (10) | 245 (6.1) | -0.1 | 244 (6.2) | 238 (6) | -0.006 |
| Concurrent medications, n (%) | | | | | | |
| ACEI&ARB | 1124 (8.6) | 200 (5) | -0.1 | 184 (4.7) | 194 (4.9) | 0.01 |
| BB | 1997 (15.3) | 711 (17.8) | 0.06 | 757 (19.1) | 702 (17.8) | -0.03 |
| CCB | 1168 (9) | 287 (7.2) | -0.06 | 236 (6) | 274 (6.9) | 0.03 |
| Anticonvulsant | 1592 (12.2) | 1128 (28.3) | 0.4 | 1153 (29.2) | 1099 (27.8) | -0.03 |
| Anxiolytic | 7170 (55.1) | 2183 (54.7) | -0.008 | 2274 (57.5) | 2155 (54.5) | -0.06 |
| ESA | 69 (0.5) | 16 (0.4) | -0.01 | 12 (0.3) | 15 (0.4) | 0.01 |
| Iron | 11 (0.1) | 10 (0.3) | 0.04 | 5 (0.13) | 8 (0.2) | 0.01 |
| Loop diuretics | 268 (2.1) | 88 (2.2) | 0.01 | 80 (2) | 81 (2.1) | 0.001 |
| Other diuretics | 675 (5.2) | 143 (3.6) | -0.07 | 126 (3.2) | 136 (3.4) | 0.01 |
| Statin | 1267 (9.7) | 317 (7.9) | -0.06 | 296 (7.5) | 307 (7.8) | 0.01 |
| AGIs | 52 (0.4) | 4 (0.1) | -0.06 | 2 (0.1) | 4 (0.1) | 0.01 |
| DPP4 inhibitors | 163 (1.3) | 77 (1.9) | 0.05 | 59 (1.5) | 70 (1.8) | 0.02 |
| GLP-1 agonists | 1 (0.01) | 8 (0.2) | 0.06 | 1 (0.03) | 0 (0) | -0.02 |
| Insulin | 225 (1.7) | 72 (1.8) | 0.006 | 51 (1.3) | 62 (1.6) | 0.02 |
| Meglitinides | 27 (0.2) | 3 (0.1) | -0.03 | 3 (0.08) | 3 (0.08) | 0 |
| Metformin | 408 (3.1) | 124 (3.1) | -0.002 | 99 (2.5) | 112 (2.8) | 0.02 |
| SGLT2 inhibitors | 28 (0.2) | 7 (0.2) | -0.009 | 8 (0.2) | 7 (0.18) | -0.005 |
| Sulfonylurea | 270 (2.1) | 50 (1.3) | -0.06 | 32 (0.8) | 45 (1.1) | 0.03 |

ACEI, angiotensin-converting enzyme inhibitor; AGIs, α-glucosidase Inhibitors; ARB, angiotensin II receptor blocker; BB, beta blocker; CCB, calcium channel blocker; COPD, chronic obstructive pulmonary disease; DM, diabetes mellitus; DPP4 inhibitors, dipeptidyl peptidase4 inhibitors; ESA, erythropoiesis-stimulating agents; ESRD, end stage renal disease; GLP-1 agonists, glucagon-like peptide 1 agonists; SGLT2 inhibitors, sodium-glucose cotransporter-2 inhibitors.

**Table S11.** Sensitivity analysis of single source inhibition constant (K_i_)

| **Model** | **R^2^ (original analysis, aggregated K_i_)** | **R^2^ (sensitivity analysis, single-study K_i_)** |
| --- | --- | --- |
| Metric 1 | 0.29 | 0.12 |
| Metric 2 | 0.61 | 0.61 |
| Metric 3^*^ | 0.28 | 0.28 |
| Metric 4^*^ | 0.95 | 0.95 |
| Metric 5 | 0.03 | 0.03 |
| Metric 6 | 0.42 | 0.46 |

R^2^, coefficient of determination.

^*^Metrics 3 and 4 are based on receptor reversal rate (K_r_) values and therefore are unaffected by the single source pK_i_ recalculation.


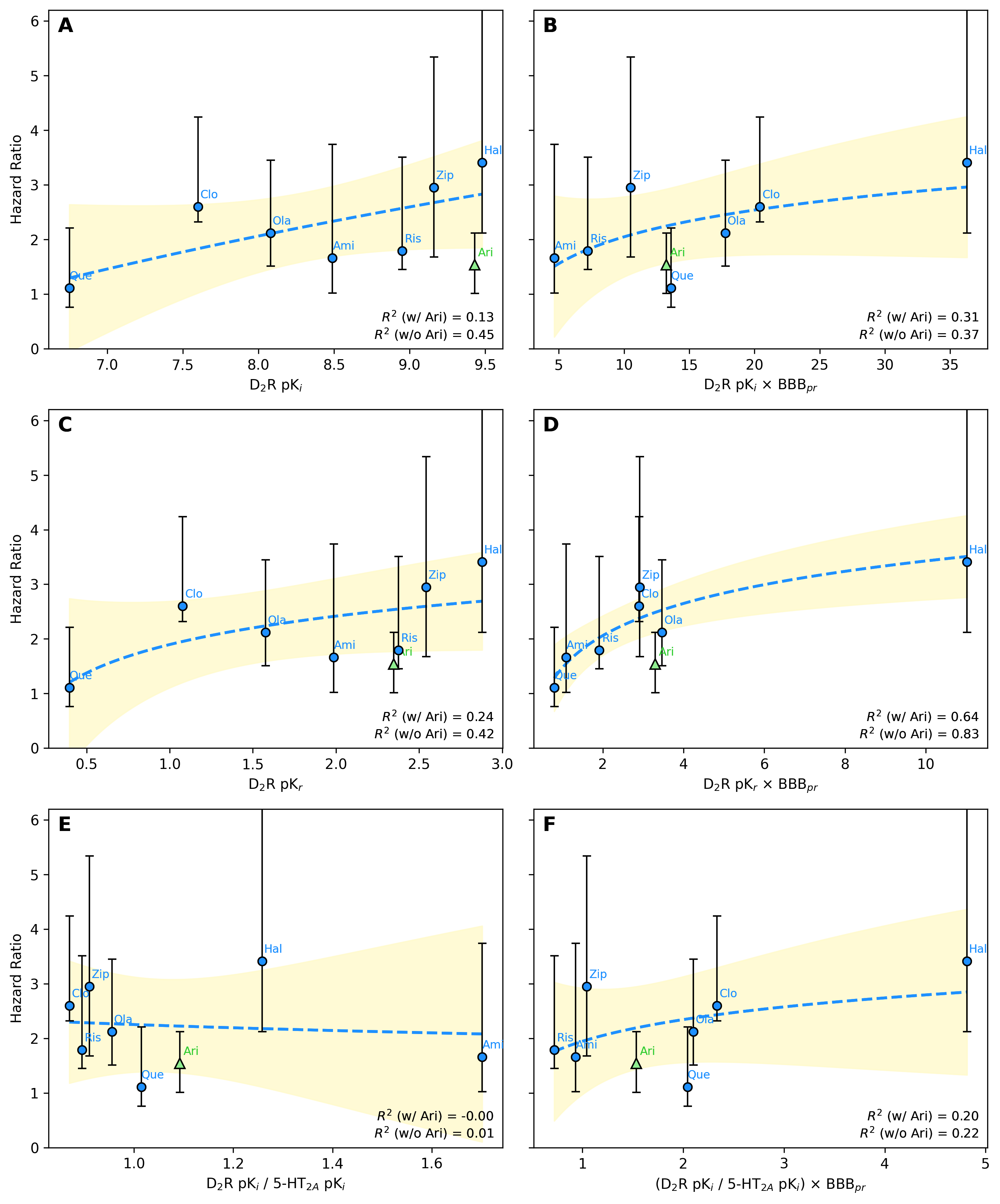


**Figure S1.** Sensitivity analysis of the outcome definition without anticholinergic confirmation

5-HT_2A_R, serotonin 2A receptor; Ami, amisulpride; Ari, aripiprazole; BBB_pr_, blood brain barrier penetration ratio; Clo, clozapine; D_2_R, dopamine D2 receptor; Hal, haloperidol; Ola, olanzapine; Ris, risperidone; Zip, ziprasidone. (A) Metric 1: D_2_R pK_i_, (B) Metric 2: D_2_R pK_i_ × BBB_pr_, (C) Metric 3: D_2_R pK_r_, (D) Metric 4: D_2_R pK_r_ × BBB_pr_, and (E) Metric 5: D_2_R pK_i_/5-HT_2A_R pK_i_, (F) Metric 6: D_2_R pK_i_/5-HT_2A_R pK_i_ × BBB_pr_. The vertical error bars represent the 95% confidence intervals of the hazard ratios. The blue dashed line and yellow-shaded region indicate the fitted logarithmic regression and 95% confidence band, respectively. The coefficient of determination (R^2^) is shown for models calculated with and without aripiprazole.
